# Coexistence of Genotypic and Temperature-Dependent Sex Determination in Pejerrey *Odontesthes bonariensis*

**DOI:** 10.1371/journal.pone.0102574

**Published:** 2014-07-18

**Authors:** Yoji Yamamoto, Yan Zhang, Munti Sarida, Ricardo S. Hattori, Carlos A. Strüssmann

**Affiliations:** 1 Graduate School of Marine Science and Technology, Tokyo University of Marine Science and Technology, Tokyo, Japan; 2 College of Fisheries and Life Science, Shanghai Ocean University, Shanghai, China; Temasek Life Sciences Laboratory, Singapore

## Abstract

In this study, we examined whether a homolog of the master sex-determining gene *amhy* of *Odontesthes hatcheri* is present and plays any role in testis determination of pejerrey *O. bonariensis*, a species otherwise known for its strong temperature-dependent sex determination (TSD). Screening of wild and laboratory-reared pejerrey for *amhy* revealed a high, although not complete linkage with phenotypic sex. The sex ratio in an *amhy* ^+/−^/*amhy* ^−/−^ full sibling progeny reared during the thermolabile period of sex determination at an intermediate temperature of 25°C was 68.7% male:31.3% female; all *amhy* ^+/−^ fish developed as males whereas about 2/3 and 1/3 of the *amhy* ^−/−^ were female and male, respectively. Expression analyses revealed that *amhy* transcription began during embryo stage and decreased by the end of sex determination period. The autosomal *amha* was present in all individuals regardless of *amhy* genotype; its expression increased significantly from the end of the same period in the gonads of all *amhy^+^* ^/−^ but only in part of the *amhy^−^* ^/−^ animals. After histological gonadal differentiation, all gonads of *amhy* ^−/−^ animals with *amha* ISH signals were testes and those without it were ovaries. These results suggest that *amhy* is important for testicular differentiation in pejerrey, at least at intermediate temperatures. Thus, we hypothesize that *amhy* ^+/−^ animals differentiate as males by expression of either *amhy* alone or *amhy* and *amha* together whereas the *amhy* ^−/−^ probably rely solely on *amha* expression. These findings represent the first clear genomic evidence that genotypic and environmental sex determinants can coexist in species with marked TSD such as the pejerrey. The finding of *amhy* will make possible to monitor wild pejerrey populations for mismatches between genotypic and phenotypic sex and may prove instrumental for field studies addressing the effects of endocrine disruptors or abnormal temperatures on reproduction and the ecological relevance of TSD for this species.

## Introduction

The pejerrey *Odontesthes bonariensis* is an excellent model for the study of temperature-dependent sex determination (TSD) in teleosts. In this species, sex ratios reach 100% female or 100% male at environmentally relevant temperatures of 17°C (female producing temperature, FPT) and 29°C (male producing temperature, MPT), respectively. The critical time of sex determination has been estimated between 1 and 5 weeks after hatching (wah) depending on the water temperature [1]. The end of this period coincides with the beginning of the histological differentiation of the gonads, which occurs first in ovaries and then in testes [2]. In addition, significant information on the molecular and biochemical processes underlying its TSD is available. For example, differential expression of *fshb* (follicle stimulating hormone beta) and *lhb* (luteinizing hormone beta) in the pituitary and of *lhr* (luteinizing hormone receptor), *cyp19a1a*, *dmrt1*, and *amh* in the gonads were found to be involved in the sex differentiation process [3]–[5]. Other studies have shown a connection between environmental temperature and sex determination that is mediated by the glucocorticoid stress-related hormone cortisol, in particular during masculinization [6], [7]. Thus, significant advances have been achieved concerning the mechanism of TSD in pejerrey but, as discussed next, the picture is far from complete.

While the reproducible sex ratios consistently obtained at the FPT (all-female) and MPT (all-male) suggest that genotypic sex determinants in *O. bonariensis* are virtually inexistent, this is not a foregone conclusion. For example, at intermediate, mixed sex-producing temperatures (MixPT; around 24–26°C), large variability in sex ratios (e.g. 20–80%) is observed between progenies from different parents at a given temperature. Such variability could be related to subtle, hitherto unknown environmental effects besides temperature or it could be an indication that parents carry some form of genotypic gender determinant that affects sex determination at sexually neutral temperatures [1]. The latter scenario has become more plausible after a recent study on the sex-determining mechanism of the congeneric species *O. hatcheri* (Patagonian pejerrey), which possesses a typically balanced (1∶1) sex ratio at intermediate temperatures, revealed a male-specific duplication of the *amh* gene (called *amhy*, for Y-linked anti-Müllerian hormone) that triggers testicular development [8]. Because the two species are closely related and share a high genetic identity [9], it is conceivable that *amhy* could exist in *O. bonariensis* and be behind the variable sex ratios observed at the MixPT, as it would be the case for example, if any of the parents is a (thermally) sex-reversed animal. It is noteworthy that environment and genotype interactions have been implied before in sex determination of other species with TSD [10]–[15] but a clear genotypic factor has never been identified.

In this context, this study was designed to probe the presence of *amhy* in the pejerrey genome and whether it has a role in gonadal sex determination of this species. We successfully cloned an *amhy* homolog in laboratory-reared pejerrey, genotyped broodstock and wild fish based on *amhy*, and carried out progeny tests to confirm its sex linkage and Mendelian inheritance. In addition, we examined the ontogeny of *amhy* expression in relation to that of the autosomal form *amha* and to time of histological gonadal sex differentiation. The results clearly show that *amhy* is functionally implicated in testicular differentiation in pejerrey at intermediate, temperatures, and prove the coexistence of environmental and genotypic sex determination in this species.

## Materials and Methods

### Ethical statement

This study was carried out in accordance with the Guide for the Care and Use of Laboratory Animals from Tokyo University of Marine Science and Technology (TUMSAT). Experiments with fish at TUMSAT do not require any special authorization as long as they adhere to the institutional guidelines, which is the case of this study. Laboratory fish were procured from the Aquatic Animal Rearing Facilities of TUMSAT, which is licensed to keep broodstock and propagate fish, and were sacrificed by anesthetic overdose in order to minimize animal suffering prior to any sampling. All samples of wild fish used in this study were a kind donation from Dr. Seiichi Kasuga, National Institute for Environmental Studies (NIES), Ibaraki, Japan and were already dead when provided to us. These samples were taken in 2001 during routine fisheries resource assessments conducted by the NIES and have been kept frozen until use. Pejerrey is not an endangered species and its collection is not subject to permit requirement.

### Cloning and sequencing of pejerrey *amhy*

To obtain the complete cDNA sequence of the *amhy* gene in *O. bonariensis*, total mRNA extracted from the gonad of a laboratory-reared, *amhy*-positive (*amhy* ^+^) was used. Extraction of mRNA and synthesis of cDNA were performed according to previous studies [8]. 5′ and 3′ UTR fragments were amplified by the primers listed in Table S1 using GeneRacer (Invitrogen, Carlsbad, CA) and Smart RACE cDNA amplification (Clontech, Mountain View, CA) kits, following manufacturer's instructions. Genomic DNA was extracted following the protocol described by Aljanabi and Martinez [16] and used for intron sequencing. PCR was performed using primers designed on the basis of the *O. hatcheri amhy* (Table S1; NCBI accession code HM153803). All amplifications were done according to the following conditions: 3 min at 94°C, 30 cycles of 30 sec at 94°C, 45 sec at 60°C and 2.5 min at 72°C, then followed by a final elongation for 5 min at 72°C. PCR products were electrophoresed in 1% agarose gel, purified, and sequenced in an ABI PRISM 3100 capillary sequencer (Life Technologies, Carlsbad, CA) using the BigDye Terminator method. Sequences were analyzed with GENETYX version 11.0 (GENETYX, Tokyo, Japan).

### Phylogenetic analysis

The predicted amino acid sequences of pejerrey Amhy and Amha (GeneBank accession numbers KC847082 and AY763406, respectively) were compared to the Amh sequences of other teleosts available at GenBank using the software GENETYX version 11.0. The following sequences were compared: Patagonian pejerrey Amhy and Amha (*Odontesthes hatcheri*, DQ441594 and HM153803, respectively), Atlantic salmon Amh (*Salmo salar*, AY722411), zebrafish Amh (*Danio rerio*, AY721604), Japanese flounder Amh (*Paralichthys olivaceus*, AB166791), blue tilapia Amh (*Oreochromis aureus*, DQ257618) and Japanese medaka Amh (*Oryzias latipes*, AB214971). The phylogenetic tree was constructed by the Neighbor-Joining method [17] using MEGA software (vers. 5.2.2) [18] with 10000 replicates.

### *amhy* genotyping of wild fish and laboratory broodstock

A random sample of 90 pejerrey juveniles collected by seine net in the Lake Kasumigaura (Ibaraki, Japan) on September 2001 and 24 laboratory-reared broodstock fish from the Aquatic Animal Rearing Facilities, Tokyo University of Marine Science and Technology (Shinagawa Campus, Tokyo, Japan), were screened for the presence of *amhy* using primers designed on the basis of the 5′ flanking region of *O. hatcheri amhy* (Table S1; NCBI accession code HM153804). The autosomal *amh* homolog of *O. bonariensis* (*amha*; NCBI accession code AY763406) was analyzed using the primers indicated in Table S1 as a positive control. Animals carrying the *amhy* gene (*amhy*-positives) were represented by *amhy* ^+^ when the exact genotype could not be determined and by *amhy* ^+/+^ or *amhy* ^+/−^ when they were confirmed as homozygous or heterozygous, respectively. Those without *amhy* (*amhy*-negative) were represented by *amhy* ^−/−^. Genomic DNA extraction and amplification followed the protocols described in the previous section. Gonadal sex of each individual was asserted by dissection and visual inspection of the gonads for wild fish, after sacrificing them through procedures described above, or manual stripping of gametes/gonadal cannulation for laboratory broodstock.

After *amhy* genotyping, laboratory-reared broodstock were used in single-pair crosses between one *amhy* ^−/−^ female and nine *amhy* ^+^ males were produced by artificial fertilization for testing Mendelian inheritance and whether the males were homozygous (*amhy* ^+/+^) or heterozygous (*amhy* ^+/−^). We also performed a progeny test with one *amhy* ^+^ female and an *amhy* ^−/−^ male. Incubation until hatching was performed as described below. Randomly-chosen hatchlings (n = 24–98) from each cross were analyzed following the same procedures used for wild fish and broodstock genotyping.

### Rearing procedures and sampling for mRNA expression analysis

One of the pairs that yielded a balanced sex ratio in the progeny test (*amhy* ^−/−^ female, F1, *amhy* ^+/−^ male, M9; Table S2) was selected and allowed to breed naturally in a 650-liter recirculated-water rearing tank under controlled temperature (20°C), photoperiod (14L/10D), and salinity (0.2–0.5% NaCl in dechlorinated tap water). Fertilized eggs were collected, cleaned of chorionic filaments, and transferred to incubators with flowing brackish water (salinity of 0.2–0.5%) at 19°C. After hatching (about 9 days after fertilization), approximately 800 to 1000 newly-hatched larvae were stocked in each of two 60-liter tanks and reared at 25°C (MixPT) [1], [2] for up to 14 weeks. Fish were fed live *Artemia* nauplii from the first day to satiation three to four times daily and gradually weaned into powdered fish food (TetraMin flakes, Melle, Germany) from the third week. Fish were sampled daily (0 to 8 days after fertilization, or daf; n = 10) and weekly (0 to 10 wah; n = 20), respectively, for gene expression and histological analyses (see below for details). Larvae and juveniles were fin-clipped for genomic DNA extraction and *amhy* genotyping according to the methods described in the previous section. The remaining fish (n = 67) were collected at the end of the experiment (14 wah) for histological determination of sex ratios.

### Histological analysis of gonadal sex differentiation and sex ratios

For the histological analysis of gonadal sex, trunks were fixed overnight in Bouin's fixative solution, dehydrated in ascending ethanol series, cleared in xylene, and embedded in Paraplast Plus (McCormick Scientific, St. Louis, MO). Cross-sections were cut serially at a thickness of 5 µm, stained with Hematoxylin-Eosin, and analyzed following previously reported histological criteria [2], [19].

### Tissue distribution and temporal expression analysis of *amhy, amha*, and *cyp19a1a* transcripts

The tissue distribution of *amhy* and *amha* transcripts was analyzed using total RNA extracted from testis, brain, gill, heart, trunk kidney, spleen, liver, anterior and posterior intestine, and muscle of an *amhy* ^+/−^ 20 week old juvenile. For the temporal expression analysis, whole embryos and trunks of larvae were stored in RNAlater (Sigma-Aldrich, St. Louis, MO) at −80°C until use. Trizol Reagent (Life Technologies) was used for total RNA extraction. Genomic DNA extracted from the remaining interphase was used for genotyping embryos. All procedures followed the reagent manufacturer's protocol. Synthesis of cDNA and transcription analyses of *amhy*, *amha*, and *β-actin* in whole embryos and juvenile tissues were performed by RT-PCR according to a previous study [8]. In larvae, the same genes were analyzed by qRT-PCR using the specific sets of primers and probes shown in Table S1. The suitability of *β-actin* as an endogenous control was confirmed by qRT-PCR in the same samples (Fig. S1, see also references [3]–[7]). The specificity of the primers was confirmed by using plasmids containing *amhy* or *amha* ORFs as controls and also by direct sequencing of PCR products. The transcript levels of the ovarian differentiation marker *cyp19a1a* were analyzed at 4 and 6 wah following methods reported in our previous studies ([4]–[6]; see also Table S1).

### Localization of *amhy/amha* mRNAs by ISH

Samples for *in situ* hybridization (ISH) in pre- and post-differentiation gonads were collected at 4 and 10 wah, fixed and processed for preparation of histological sections as described above. Body trunk sections were hybridized in the automated tissue processer Hybrimaster HS-500 (Aloka, Tokyo, Japan) using an *amh* probe that recognizes both *amhy* and *amha*, synthesized according to a previous study [5]. Final detection was performed manually with NBT/BCIP according to the manufacturer's (Roche Diagnostics, Basel, Schweiz) protocols.

## Results

### Cloning and sequence analysis of *amhy* gene

**Figure 1:**
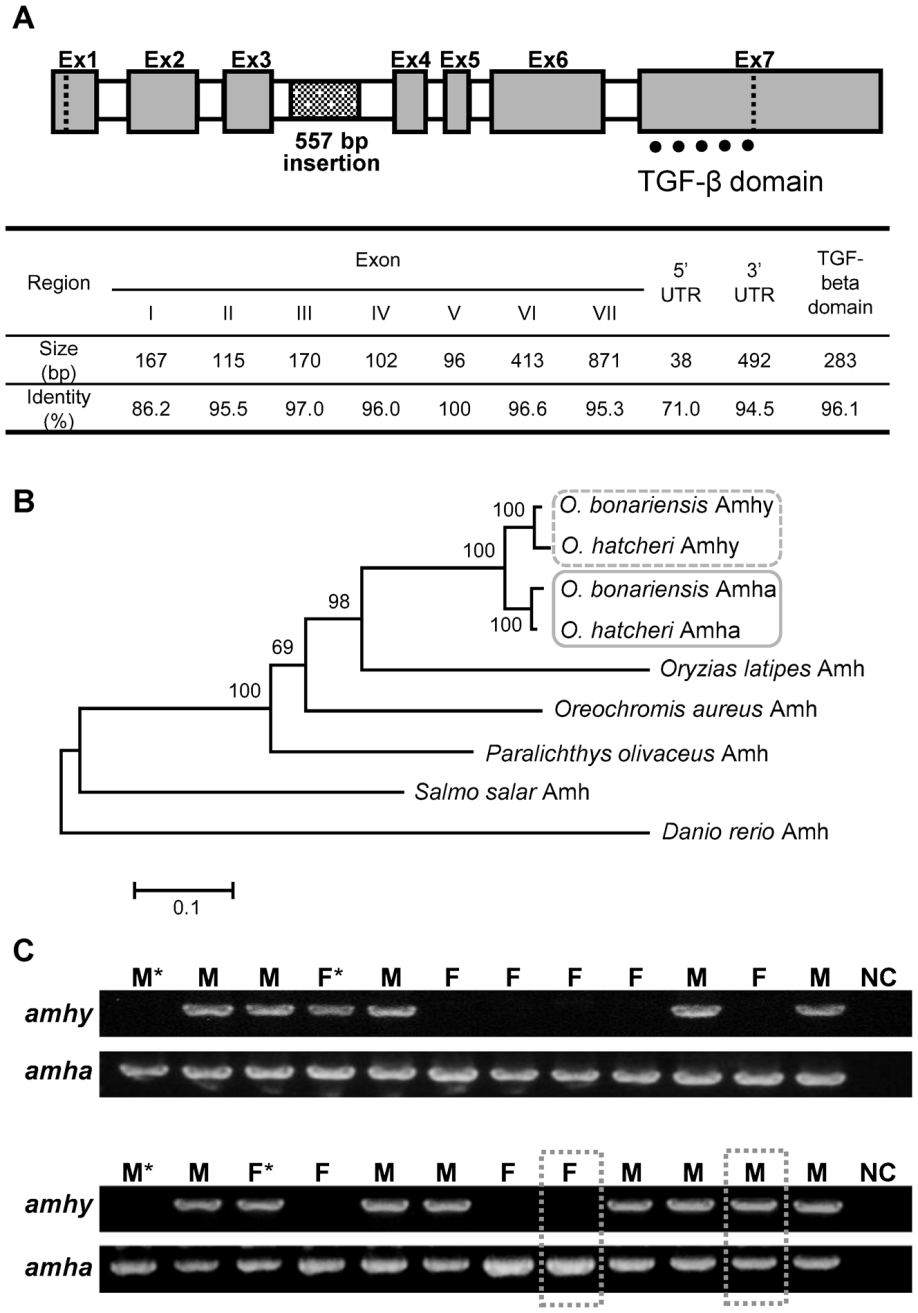
*amhy* gene structure, phylogenetic relationship, and broodstock genotyping. **A**: Structure of the *amhy* gene in *O. bonariensis*, size of exons, UTRs, and TGF-beta domain, and the respective identity values in relation to *O. bonariensis amha*. The third intron contains a 0.5 kb insertion in relation to *amha*. **B**: Phylogenetic tree (Neighbor-Joining method) for the amino acid sequences of *O. bonariensis* and *O. hatcheri* Amhy and Amha and the Amh of other teleosts. Numbers indicate bootstrap values based on 10000 replicates. **C**: *amhy*-based sex genotyping in *O. bonariensis* broodstock using primers that amplify part of the 5′ flanking region and part of the *amhy* gene (1896 bp); *amha* gene was used as positive control (2441 bp). The dotted-boxes indicate parents used in the rearing experiment and asterisks indicate disagreement between the *amhy*-based genotype and phenotypic sex. NC: negative control.

An *amhy* homolog was cloned from a laboratory-reared pejerrey and revealed the *amhy*-characteristic 0.5 kb fragment within the third intron (Fig. 1A). The deduced Amhy protein, including the characteristic TGF-β domain (amino acids 421–514) with seven canonical cysteine residues, comprised 514 amino acids. Phylogenetic analysis based on the amino acid sequence of the open reading frame showed that *O. bonariensis* Amhy shared the same clade with *O. hatcheri* Amhy whereas the Amha in both species were placed together in another clade (Fig. 1B). Among the outgroup species, the medaka Amh showed to have the shortest genetic distance to the *Odontesthes* species Amhs, displaying similar distances to both Amhy and Amha clades.

### Genotyping of wild fish, broodstock, and progeny from specific crosses

The analysis of juveniles from Lake Kasumigaura revealed 38 *amhy* ^+^ and 52 *amhy* ^−/−^ out of 90 individuals whereas that of our *O. bonariensis* broodstock revealed 14 *amhy* ^+^ and 10 *amhy* ^−/−^ out of 24 individuals (Table 1; Fig. 1C). In both cases, there was a high but not complete concordance between genotypic and phenotypic sex. The progeny of all 9 *amhy* ^+^ males crossed pairwise with the same *amhy* ^−/−^ female showed sex ratios statistically undistinguishable from 1∶1 (Fisher's exact test), indicating that all males were heterozygous (*amhy* ^+/−^) for the *amhy* gene (Table S2). No *amhy* ^+/+^ male was found among the tested fish. Likewise, the cross of an *amhy* ^+^ female with an *amhy* ^−/−^ male confirmed that the former was heterozygous for *amhy* (Table S2). As expected, *amha* was detected in all fish regardless of phenotypic sex or *amhy* genotype (Fig. 1C).

**Table 1:** Relationship between phenotypic (gonadal) sex and *amhy* genotype in wild pejerrey and laboratory-reared broodstock.

Source	Genotype	Phenotype		
		Female	Male	Total n (%)
Wild fish^1^^,^ 2 (Lake Kasumigaura)	*amhy* ^−/−^	49	3	52 (57.8)
	*amhy* ^+^	1	37	38 (42.2)
	Total n (%)	50 (55.6) *	40 (44.4)	
Laboratory broodstock^1^^,^ 2	*amhy* ^−/−^	8	2	10 (41.7)
	*amhy* ^+/−^	2	12	14 (58.3)
	Total n (%)	10 (41.7)	14 (58.3)	

1 No statistical significance difference in phenotypic sex ratio (Fisher's test, p>0.05).

2 No statistical significance difference in *amhy* genotype ratio (Fisher's test, p>0.05).

### Tissue distribution and temporal expression analysis of *amhy*, *amha*, and *cyp19a1a*

**Figure 2:**
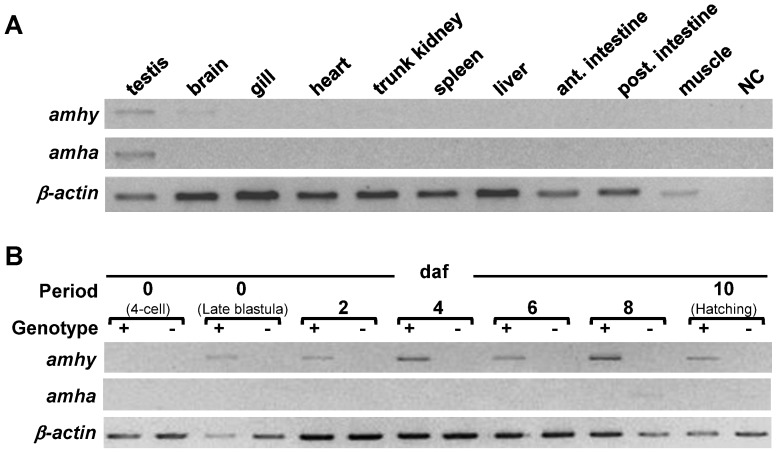
Expression of *amhy* and *amha* mRNAs in tissues and embryos. **A**: Tissue distribution of *amhy* and *amha* mRNAs in juvenile pejerrey (RT-PCR). **B**: Expression profile of *amhy* and *amha* during embryogenesis in *amhy* ^+/−^ and *amhy* ^−/−^ genotypes (RT-PCR). *β-actin* was used as endogenous control. NC: negative control.

**Figure 3:**
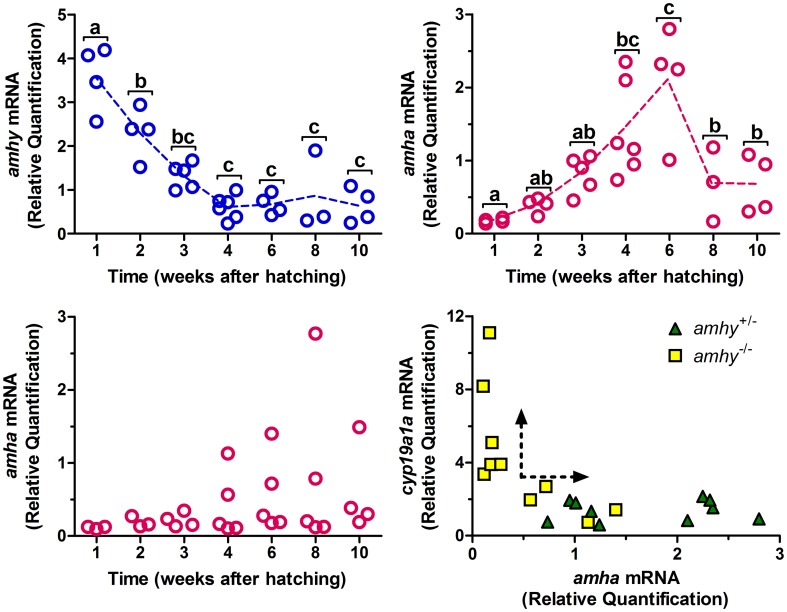
Quantification of *amhy*, *amha* and *cyp19a1a* mRNAs during sex differentiation. **A** to **C**: Abundance of mRNA transcripts of *amhy* (A) and *amha* (B) in *amhy^+/−^* genotypes and of *amha* in *amhy* ^−/−^ genotypes (C) during larval development at 25°C (n = 3 to 6 per time point; qRT-PCR). **D**: Abundance of *amha* mRNA transcripts in relation to *cyp19a1a* in *amhy* ^+/−^ and *amhy* ^−/−^ genotypes at 4 and 6 weeks after hatching (qRT-PCR); arrows indicate two arbitrarily-defined, opposing patterns of gene expression. *β-actin* was used as endogenous control. Values with different letters are statistically different from one another (One-Way ANOVA with Bonferroni's post-test, p<0.05).

Transcripts of *amhy* were found in the testis and in the brain whereas *amha* was expressed only in the testis of juveniles (Fig. 2A). Transcripts of *amhy* were detected in embryos from late blastula stage until hatching in all *amhy^+/−^* individuals (Fig. 2B). In larvae trunks, the expression of *amhy* was highest at 1 wah and decreased until 4 wah, when it reached a low but stable plateau (Fig. 3A). *amha* mRNA expression was undetectable in *amhy^+/−^* embryos (Fig. 2B) and low in larvae between 1 and 3 wah (Fig. 3B) but clearly upregulated between 4 and 6 wah. *amha* mRNA expression was not detected in any of the *amhy* ^−/−^ embryos (Fig. 2B) and was consistently low in larvae between 1 and 3 wah (Fig. 3C). In contrast, between 4 and 10 wah the mRNA expression assumed a bimodal distribution thereby 7 out of 19 *amhy* ^−/−^ individuals (37%) had high values and the remaining ones low levels (Fig. 3C).

A comparative analysis between the expression of *amha* and the ovarian differentiation marker *cyp19a1a* at 4 and 6 wah revealed that all 10 *amhy^+/−^* individuals had high and low transcript levels of *amha* and *cyp19a1a*, respectively (Fig. 3D). The *amhy* ^−/−^ animals, on the other hand, showed either this pattern (4 out of 10 individuals) or the opposite one with relatively high *cyp19a1a* and low *amha* levels (6 out of 10 individuals; Fig. 3D).

### Localization of *amha/amhy* mRNAs by ISH

**Figure 4:**
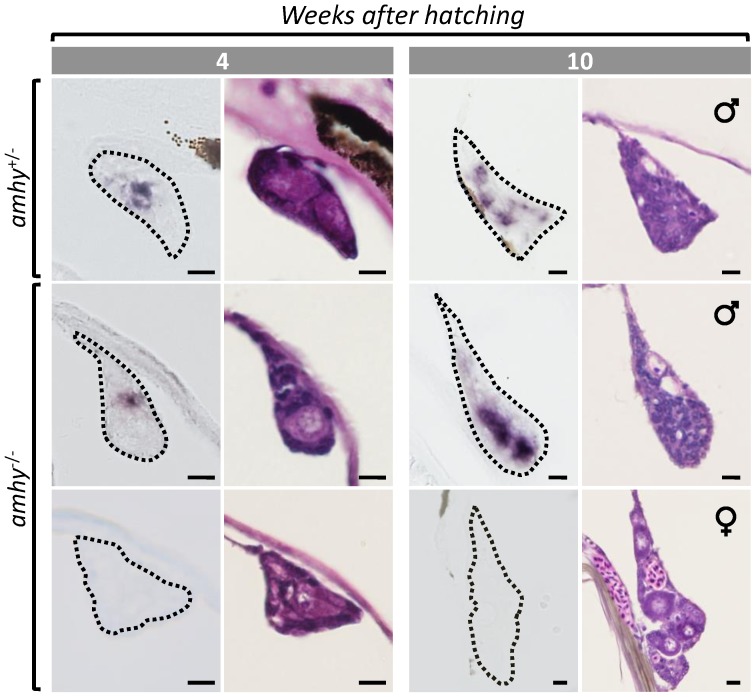
Spatial expression of *amhy* and *amha* mRNAs in differentiating gonads. Localization *amhy* and/or *amha* transcripts by ISH (left panels) and light microscopic histology (right panels) of gonads in 4 and 10 week old larvae reared at 25°C. Transcripts were detected in all *amhy* ^+/−^ genotypes (presumptive *amhy* and/or *amha* signals) and in about half of the *amhy* ^−/−^ genotypes (*amha* signals). At 10 wah, the expression was detected in developing testis but not in developing ovaries. Scale bars indicate 10 µm.

ISH signals for *amha/amhy* were detected exclusively in somatic cells of the medullary region of gonads of all *amhy* ^+/−^ (n = 2 for each sampling point) and in 8 out of 14 *amhy* ^−/−^ individuals from 4 and 10 wah (Fig. 4). At 10 wah, when all gonads had differentiated as ovaries or testes, only the latter had ISH signals.

### Relation of phenotypic sex to amhy genotype under controlled conditions

The remaining fish from the *amha*/*amhy* expression analysis at 14 wah (n = 67) were 68.7% males and 31.3% females. The ratio of *amhy* ^+/−^ to *amhy* ^−/−^ fish was nearly 1∶1 (49.3%:50.7%) and all of the formers (n = 33) were phenotypically male. Among the 34 *amhy* ^−/−^ fish, 21 (61.8%) and 13 (38.2%) were female and male, respectively. The gonads of all individuals examined, including the testes of both *amhy* ^−/−^ and *amhy* ^+/−^ males, had no abnormalities or difference of any kind compared to previously reported criteria [2], [19] (data not shown).

## Discussion

In this study, we examined whether a homolog of the sex determining gene *amhy* of *Odontesthes hatcheri* [8] is present and plays any role in testis determination of pejerrey *O. bonariensis*, a species otherwise known for its strong temperature-dependent sex determination [1]. Cloning of the *O. bonariensis amhy* revealed a molecule that is 98% and 97% identical in terms of the open reading frame and TGF-β domain, respectively, to its homolog in *O. hatcheri*. Wild-caught pejerrey and captive broodstock were then genotyped on the basis of *amhy*, showing its presence in about half of the individuals and, for those that were phenotypically sexed, with few exceptions, they were males. More importantly, *amhy^+^* ^/−^ was linked 100% to maleness in a progeny that was reared throughout the critical period of sex determination under a temperature (25°C) known to produce mixed-sex populations [1], [2]. Conversely, most of the *amhy^−/−^* individuals were females although there were clearly more exceptions among those reared at 25°C (e.g., approximately 1/3 of *amhy^−/−^* males; see further discussion below about the effects of this temperature). In this context, and keeping in mind the strong effects of water temperature on pejerrey sex determination [1], the results suggest that *amhy* is sex-linked in *O. bonariensis* and that it could be implicated in the sex determination of this species just as it is in *O. hatcheri* [8].

To address this hypothesis, we examined the ontogeny of *amhy* expression during gonadal sex determination and histological sex differentiation in offspring from an *amhy* ^−/−^ female and an *amhy^+/−^* male raised under controlled laboratory conditions. During incubation at 19°C, *amhy* transcripts were consistently expressed from the late-blastula stage onwards in all *amhy^+/−^* genotypes. The *amhy* transcription was maintained through hatching and transfer to 25°C, the period considered as critical for sex determination (1–5 wah) [1], and finally the appearance of histological signs of gonadal differentiation (4–7 wah) [2]. This pattern of expression is consistent with a role in gonadal differentiation and, considering its sex linkage, the cellular pattern of expression described below, as well as the known involvement of Amh in testicular differentiation in several fish species including its congener *O. hatcheri* [4], [8], [20], with testicular development. Still, the expression from early embryogenesis, even before the formation of the gonad anlagen, is intriguing. This is much earlier than in *O. hatcheri* where *amhy* plays the master trigger for testicular differentiation [8]. Whether this early sex-specific expression can affect sex afterwards by epistatic effects on other genes, hence predisposing the *amhy^+/−^* genotypes to become males, remains to be assessed. Other questions concerning *amhy* that must be addressed are to what degree its expression is affected by water temperature, if it acts through or independently of *amha* (see the following discussion), and if the expression found in the brain is implicated in sex differentiation.

In contrast to *amhy*, *amha* was found in all fish regardless of gonadal phenotype, indicating that it is located in autosomal chromosomes just as it is in *O. hatcheri* [8]. Yet, it seems to be critical for masculinization in *amhy^−/−^* individuals, perhaps as a function of temperature and endocrine factors [4], and may be a coadjuvant factor in *amhy^+/−^* genotypes. The first line of evidence that supports a role for *amha* is that its expression, although not as early as that of *amhy*, coincided temporally with the period when the pejerrey gonads are still sexually labile (see references above). This pattern differs from the late *amha* expression described in *O. hatcheri*, where it is considered as irrelevant for testicular differentiation [8]. Further, both qRT-PCR and ISH revealed a bimodal pattern of *amha* expression in *amhy* ^−/−^ individuals where the proportion of animals with high *amha* expression during the estimated period of sex determination (37%) closely approximated the proportion of animals with low *cyp19a1a* during the same period (40%) and that of phenotypic males determined at 14 wah (38%). Also, when the gonads had clearly differentiated by 10 wah, gonads showing *amha* expression were testes whereas those without it were ovaries. Finally, all *amhy^+/−^* animals had high *amha* as well as low *cyp19a1a* transcription during the period of sex determination and all became males.

Taken together, these results strongly suggest that *amhy* ^+/−^ genotypes differentiate as males by expression of either *amhy* alone or *amhy* and *amha* together and that *amhy* may be implicated in the up regulation of *amha*. We also hypothesize that *amhy* ^−/−^ genotypes rely on *amha* expression for testis differentiation. Nevertheless, the actual processes underlying *amha* regulation in both genotypes remain to be elucidated. In this regard, it must be noted that the TGF-beta domain, the region that binds to the primary receptor AmhrII, is highly conserved in both *amhy* and *amha* genes of *O. bonariensis* as in *O. hatcheri* [8]. Thus, we suppose that Amha may activate the same AmhrII used by Amhy for the activation of downstream pathway of testis differentiation in *amhy* ^−/−^ genotypes. Ongoing studies are focusing on the thermal thresholds for mRNA expression, receptor binding, and the relative contributions of *amhy* and *amha* for masculinization.

The sex ratio in the controlled rearing experiment was significantly (about 70%) male-biased and only female-to-male sex-reversals were noted. This highlights the importance of the discovery of *amhy* for unbiased and accurate screening of thermal effects on gonadal sex differentiation. Thus, the current results suggest that 25°C might not be exactly neutral for pejerrey in terms of sex effects as previously assumed [1]. Alternatively, other forms of stress may have caused elevation in cortisol levels, which is able to induce testicular differentiation [6], [7], and thus activated the male pathway leading to sex-reversal. Given the results obtained in this study, it could be argued that pejerrey possesses a genotypic sex determinant in spite of having a marked TSD. This finding underscores the difficulty in drawing a line between GSD and TSD and that these forms are likely part of a continuum [21], [22]. On the other hand, it is intriguing how *amhy* has been maintained in the course of evolution in a species whose sex is highly susceptible to temperature effects. The high thermal dependence of sex associated to the presence of a marker for genetic predisposition of gender makes *O. bonariensis* a very attractive model to study these issues as well as the molecular pathways of high temperature-induced masculinization and low temperature-induced feminization. Although in low frequency, both *amhy* ^+/−^ females and *amhy* ^−/−^ males were found in a wild population, raising concerns about its causes and the impact of temperature-dependent sex determination and sex-reversals on the population demographics [12]. The finding of *amhy* will make possible to monitor wild pejerrey populations for mismatches between genotypic and phenotypic sex and may prove instrumental for field studies addressing the effects of endocrine disruptors or abnormal temperatures on reproduction and the ecological relevance of TSD for this species.

In summary, this study demonstrated that the *amhy* gene is active in *amhy^+/−^* genotypes before, during, and after the critical time-window of TSD. Although some *amhy^−/−^* individuals developed as males, no *amhy* ^+/−^ females were found among fish reared at intermediate temperatures, suggesting that under similar conditions *amhy* is a strong determinant of testis differentiation. Taken together, the present results provide strong support for the coexistence of GSD and TSD in *O. bonariensis*.

## Supporting Information

Figure S1 **Quantification of** ***β-actin*** **mRNA during larval development.** Abundance of *β-actin* mRNA transcripts in trunks of larvae reared from 1 to 10 weeks after hatching at 25°C (qRT-PCR). Symbols and bars indicate the means and SEM, respectively. Values with the same letter are not statistically different from one another (One-Way ANOVA with Bonferroni's post-test, p>0.05).
(TIF)

Table S1 **Details of the primers used for** ***amhy*** **cloning,** ***amhy*** **genotyping and expression analysis with the respective PCR conditions.**
(DOCX)

Table S2 **Proportion of** ***amhy*** **^+^ and** ***amhy*** **^−/−^ genotypes in the progenies produced by single-pair crosses using laboratory broodstock fish.**
(DOCX)
