# Supplementary material for: Coexistence of Genotypic and Temperature-Dependent Sex Determination in Pejerrey Odontesthes bonariensis
Source: PLoS One. 2014 Jul 18;9(7):e102574. doi: 10.1371/journal.pone.0102574 (PMC4103838; doi:10.1371/journal.pone.0102574)
Supplement: Table S1 — Details of the primers used for amhy cloning, amhy genotyping and expression analysis with the respective PCR conditions. (DOCX) [file pone.0102574.s002.docx]

| **Purpose** | **Oligo name** | **Oligo sequence (**’**5 –** ’**3)** | **Primer binding sites (amplicon size)** | **PCR conditions** |
| --- | --- | --- | --- | --- |
| *amhy* UTRs  amplification | Amhy 3RACE-1st  Amhy 3RACE-2nd:  amhy 5RACE-1st  amhy 5RACE-2nd: | AGTCTCACCGTGTCCTTCGAAAAG GGTGGTCATAGACTGGAACGAGGAA  CAGAAGACGCCATATTGAGAC TCCACAAAGCAGGGTATGAG | +1276 to +1299  +1464 to +1488  +1905 to +1925  +142 to +161 | 1x 94°C 2 min; 35x 94°C 30 sec, 58°C 30 sec and 72°C 90 sec; 72°C 2 min 1x 94°C 2 min; 35x 94°C 30 sec, 56°C 30 sec and 72°C 90 sec; 72°C 2 min |
| *amhy* amplification  (*amhy* genotyping) | OhaYPFw  OhaYPRv | AGTCAGCTCAGATGCT  AGCCGGATGCAAAACTTCCAGA | -1387 to -1371  +317 to +339  (1896 bp) | 1x 94°C 5 min; 35x 95°C 30 sec, 60°C 30 sec, 72°C 150 sec; 1x 72°C 5 min |
| *amha amplification* | OboAmhaFw  OboAmhaRv | ACGCGGGTCACACAGGCGTTTC  CCGTCTGCATAAAACAAAC | -38 to -17  +1194 to +2394  (2432 bp) | 1x 94°C 5 min; 35x 95°C 30 sec, 60°C 30 sec, 72°C 150 sec; 1x 72°C 5 min |
| *amhy* qRT-PCR  (TaqMan) | qPCR-amhyFw  qPCR-amhyRv  TaqMan Probe | GCACGTCGGAGGTCGGA  GAGGTTATGAGGTGCTGAGGAAGTTA  TCGTGCATCGGCAGAG | -38 to -21  +118 to +144  +53 to +69  (182bp) | 1x 50°C 2 min; 1x 95°C 20 sec; 40x 95°C 3 sec, 63°C 30 sec |
| *amha* qRT-PCR  (Taqman) | qPCR-amhaFw  qPCR-amhaRv  TaqMan Probe | AAACAGCAGCAGGTGAGAGTCA  TGATGGAGAGAAAAGACTCTTCCG  CCAGTCCACGACCTCCAGGGGGT | +1130 to +1151  +1511 to +1534  +1447 to +1469  (405bp) | 1x 50°C 2 min; 1x 95°C 20 sec; 40x 95°C 3 sec, 60°C 30 sec |
| *β-actin* qRT-PCR  (TaqMan) | qPCR-actinFw  qPCR-actinRv  TaqMan Probe | TCGTGCGCGACATTAAGGA  GCAGCGGTCCCCATTTC  CTGTGTTACGTTGCATTGGACTTTGAGCA | +623 to +641  +676 to +692  +646 to +674  (70bp) | 1x 50°C 2 min; 1x 95°C 20 sec; 40x 95°C 3 sec, 60°C 30 sec |
| *β-actin* qRT-PCR  (SYBR Green) | qPCR-obb actinFw  qPCR-obb actinRv | GCTGTCCCTGTACGCCTCTGG  GCTCGGCTGTGGTGGTGAAGC | +417 to +437  +596 to +616  (200bp) | 1x 50°C 2 min; 1x 95°C 10 min; 40x 95°C 10 sec, 60°C 30 sec |
| *cyp19a1a* qRT-PCR  (SYBR Green) | qPCR-AromGFw  qPCR-AromGRv | GCGAGCTGTCTGGCTGAGAA  AGGAGCAGCAGCATGAAGAAGA | +902 to +920  +980 to +1001  (200bp) | 1x 50°C 2 min; 1x 95°C 10 min; 40x 95°C 10 sec, 60°C 30 sec |
