# Supplementary material for: Coexistence of Genotypic and Temperature-Dependent Sex Determination in Pejerrey Odontesthes bonariensis
Source: PLoS One. 2014 Jul 18;9(7):e102574. doi: 10.1371/journal.pone.0102574 (PMC4103838; doi:10.1371/journal.pone.0102574)
Supplement: Table S2 — Proportion of amhy + and amhy −/− genotypes in the progenies produced by single-pair crosses using laboratory broodstock fish. (DOCX) [file pone.0102574.s003.docx]

| **Crosses** | | **Progeny**  **(n)** | **Genotype Proportion** | |
| --- | --- | --- | --- | --- |
| **Female**  **(genotype)** | **Male**  **(genotype)** |  | ***amhy*^+^** | ***amhy*^-/-^** |
| F1 (*amhy*^-/-^) | M1 (*amhy*^+^) | 36 | 47.2 | 52.8 |
|  | M2 (*amhy*^+^) | 50 | 50.0 | 50.0 |
|  | M3 (*amhy*^+^) | 94 | 48.7 | 51.3 |
|  | M4 (*amhy*^+^) | 81 | 47.5 | 52.5 |
|  | M5 (*amhy*^+^) | 98 | 53.1 | 46.9 |
|  | M6 (*amhy*^+^) | 24 | 33.3 | 66.7 |
|  | M7 (*amhy*^+^) | 56 | 51.8 | 48.2 |
|  | M8 (*amhy*^+^) | 49 | 48.9 | 51.1 |
|  | M9 (*amhy*^+^) | 30 | 53.3 | 46.7 |
| F2 (*amhy*^+^) | M10 (*amhy*^-/-^) | 35 | 40.0 | 60.0 |
